# Regional Anesthesia in Neuroanesthesia Practice

**DOI:** 10.15190/d.2020.8

**Published:** 2020-06-29

**Authors:** Ashutosh Kaushal, Rudrashish Haldar

**Affiliations:** ^1^Department of Anaesthesiology, All India Institute of Medical Sciences (AIIMS), Rishikesh, India; ^2^Department of Anaesthesiology, Sanjay Gandhi Post Graduate Institute of Medical Sciences (SGPGIMS), Lucknow, India

**Keywords:** Analgesics, anaesthetics, anaesthesia, carotid endarterectomy, cervical plexus block, neurosurgery.

## Abstract

Regional anesthesia has been an undervalued entity in neuroanesthetic practice. However, in the past few years, owing to the development of more advanced techniques, drugs and the prolific use of ultrasound guidance, the unrecognised potential of these modalities have been highlighted. These techniques confer the advantages of reduced requirements for local anesthetics, improved hemodynamic stability in the intraoperative period, better pain score postoperatively and reduced analgesic requirements in the postoperative period. Reduced analgesic requirement translates into lesser side effects associated with analgesic use. Furthermore, the transition from the traditional blind landmark-based techniques to the ultrasound guidance has increased the reliability and the safety profile. In this review, we highlight the commonly practised blocks in the neuroanesthesiologist’s armamentarium and describe their characteristics, along with their individual particularities.

## SUMMARY

*1. Introduction*

*2. Requirement of regional techniques in neuroanesthesia*

- *2.1. Blocks used in head and neck surgeries*
  - *2.1.1 Scalp block*
  - *2.1.2 Infraorbital block (IOB)*
  - *2.1.3 Trigeminal Nerve Block*
  - *2.1.4 Cervical plexus block (CPB)*

- * 2.2. Blocks used for spinal surgeries*
  - *Erector Spinae Block (ESP)*

*3. Conclusion*

## 1. Introduction

Neurosurgical Anesthesiology is a relatively modern subspecialty of anesthesiology, which focusses on the anesthetic management of patients undergoing neurosurgical procedures. Maintenance of cerebral and spinal cord perfusion pressure is the foremost consideration for neurosurgical procedure, which depends upon the maintenance of hemodynamic stability and changes in the pain intensity at different stages of surgery^1^. These procedures are usually prolonged, thus maintaining the same surgical position is difficult, even if the patient is awake or sedated with adequate analgesia. As the surgery is being conducted within narrow anatomical corridors and with high precision, slightest degree of movement by the patient is unacceptable and potentially deleterious.

In the face of these concerns, maintenance of general anesthesia with inhalational or intravenous agents have been the traditional anesthetic modality for neurosurgical patients. Regional anesthesia solely has not been proven adequate during neurosurgical procedure as compared to some other surgical specialities and, therefore, has been often disregarded or ignored. In this regard, application of regional nerve blocks when combined with general anesthesia during neurosurgery has been seen to provide hemodynamic stability, to decrease anesthetic requirement, as well as extend postoperative analgesia and, therefore, is desired.

In this review article, we highlight the possible types and techniques of regional anesthesia, which can be applied perioperatively as an adjunct to general anesthesia and discuss their benefits when administered during various types of neurosurgical procedures. In addition to the authors own experience, a systematic literature search and analyses was performed by using search engines, including the ones provided by PubMed, Google and Google Scholar, with the use of the following single-text words and combinations: anesthesia/anaesthesia, neurosurgery, regional anesthesia/anaesthesia, nerve blocks and other combinations of words, from the year 2000 to 2020. The references of relevant articles were cross-checked and the articles containing all these keywords were thoroughly studied for the development of this review.

## 2. Requirement of regional techniques in neuroanesthesia

Neurosurgical procedures are broadly classified into cranial and spinal procedures. Contemporary cranial procedures emphasise the use of functional and minimally invasive procedures, with high degree of emphasis on availability of optimal operative conditions, preservation of neurocognitive function, minimizing interference with electrophysiological monitoring, and a rapid, high-quality recovery. Small craniotomies, intraoperative imaging, stereotactic interventions, and endoscopic procedures to increase surgical precision and minimize trauma to normal tissues is prioritized. Outcome measures, such as quicker recovery, minimal perioperative morbidity, and reduced hospital stay, are desired^2^. Spinal procedures are usually accompanied by neurophysiological monitoring, which curtails the type and dosage of general anesthetic drugs. Spinal surgeries are also generally associated with intense pain in the postoperative period, especially for the initial few days. In this scenario, adequate pain management using regional anesthetic techniques correlates well with improved functional outcome, early ambulation, early discharge, and preventing the development of chronic pain^3^. Thus, appropriate application of regional anesthetic modalities facilitates intraoperative conduct, as well as improves the postoperative outcomes of neurosurgical patients. For the purpose of this review, we have broadly classified the different modalities under the headings of blocks specific for head and neck surgeries and blocks utilized in spinal surgeries. Table 1 depicts the summary of different studies related to regional neve blocks used for neurosurgery.

### 2.1 Blocks used in head and neck surgeries

#### 2.1.1 Scalp block

It consists of blocking six nerves that provide the sensory innervation of the scalp, on either side of the scalp, by subcutaneous infiltration of 2-3 ml local anesthetics (LA) for each nerve. These nerves are the supraorbital, supratrochlear, zygomaticotemporal, auriculotemporal, lesser occipital and greater occipital nerves. Usually bilateral blocks are placed. LA, such as bupivacaine, ropivacaine or levobupivacaine, are commonly used. Though the landmark technique is popular, the advent of ultrasound guidance has increased the precision of block administration. Ultrasound guidance can be used to locate the supraorbital notch (for supraorbital nerve), pterygopalatine fossa (for zygomaticotemporal nerve), superficial temporal artery (for auriculotemporal nerve) and occipital artery (for greater occipital nerve).

The main indication of scalp block is awake craniotomy. Other indications are deep brain stimulation and stereotactic radiosurgery, burr hole drainage of chronic subdural haemorrhage and cranioplasty surgery^1^. For other craniotomies, the promising advantage offered by scalp block is the capability to perform accurate neurological evaluation in postoperative period, as it does not affect other motor or sensory systems and provides pre-emptive analgesia. Benefits of an appropriately placed scalp block are present during all stages of the surgery. Preoperatively, scalp blockade blunts the hemodynamic response to cranial fixation^4^. Intraoperatively, scalp block (with bupivacaine) has been proven to be superior over control group (with saline) in terms of hemodynamic stability and decreased anesthetic requirement during cranial fixation^5,6^. Scalp block (with bupivacaine) has been found to be better compared to intravenous opioid analgesic and bupivacaine infiltration at each pin insertion site in controlling hemodynamics during cranial fixation and for 3 minutes later. Lower levels of cortisol and adrenocorticotropic hormone at different time point after cranial fixation was also demonstrated in scalp block group^7^. Effect of scalp block persists until the time of incision and until dural opening. Scalp block was found superior over control group until dural opening, but following that, the scalp block was comparable to control group with respect of hemodynamic stability^8^.

Scalp block’s effect extends into the postoperative period too and it has proved to decrease the incidence and severity of postoperative pain in patients undergoing supratentorial craniotomy^9,10^. It also reduces the incidence of requests for rescue analgesics, increases the duration between surgery completion and first demand of analgesics, and reduces pain scores in the early postoperative period^11^.

Adjuvants like opioids, dexmedetomidine^12^, dexamethasone and magnesium sulphate^13^have been investigated by different investigators for their effect on the improvement of the quality and duration of the block with varying results. Meta-analysis of different randomised control studies (RCTs) have demonstrated a consistent reduction of pain severity^14^, which extends 6 hours after craniotomy^15^. A moderate reduction in opioid consumption in the 24 hours following craniotomy is also observed^16^. Additionally, it reduces the incidences of postoperative nausea and vomiting, which is usually associated with opioid usage for analgesia^15^.

#### 2.1.2 Infraorbital block (IOB)

The infraorbital nerve is a pure sensory nerve and is a terminal branch from the second maxillary division of the trigeminal nerve that exits the skull through the foramen rotundum to enter the pterygopalatine fossa. It exits the cranium through the infraorbital foramen in a caudal and medial direction and divides into several sensory branches: the inferior palpebral, the lateral nasal, and the superior labial nerves. It supplies the skin and mucous membrane of the upper lip and lower eyelid and the cheek between them and to the lateral side of the nose.

In addition to infraorbital nerve that emerges on to the face, the IOB also blocks anterior superior alveolar and middle superior alveolar nerves that originate within the infraorbital canal and supplies the mucous membrane of the lateral wall, floor of the nasal cavity and also the nasal septum^17,18^.

Endoscopic trans-nasal trans-sphenoidal (TNTS) approach is a commonly performed procedure for pituitary tumor excision. Although it is a type of minimally invasive neurosurgery, due to sub-mucosal nasal dissection and nasal packing, patient may experience significant pain and discomfort in the postoperative period. Bilateral infraorbital nerve block combined with general anesthesia is proved to be beneficial in rapid, smooth, pain free emergence from anesthesia, facilitating quick neurological evaluation, in addition to decreasing postoperative pain and discomfort^19^.

The block can be applied using the classic anatomical approach or the ultrasound guided approach. For the classical landmark techniques, two approaches, either the intraoral or extraoral approaches, can be used. The important landmark is the infraorbital foramen, which is located just below the orbital margin, at the junction of a vertical line drawn in line of the centre of the pupil and a horizontal line from the nasal alae. In intraoral approach, the incisor and the first premolar are palpated. A 25-27-gauge needle is inserted into the buccal mucosa in the subsulcal groove at the level of the canine or the first premolar and guided upward and outward into the canine fossa. Keeping a finger over the infraorbital foramen to assess the suitable position of the needle tip and to prevent injury of the eyeball by unintentional cephalad progression of the needle into the orbit, the needle is advanced^20^. Then, after negative aspiration, 1–3 ml of local anesthetic is administered.

In extraoral approach, the infraorbital foramen is palpated, a 25-27-gauge needle is inserted perpendicularly in upward and medial direction toward the foramen, until bony resistance is felt. A finger is continuously positioned at the level of the infraorbital foramen. Then, after negative aspiration, 1–3 ml of the local anesthetic is administered.

The same block can be attempted under ultrasound guidance. Using a 6-13 Hz linear probe placed on the cheek just lateral to the nose horizontally and moving from medial to lateral direction, until a disruption is appreciated in the hyperechoic line which represents the infraorbital foramen. The relationship of the nerve with infraorbital artery is confirmed using the Doppler mode. A 23-25 G block needle is inserted using in plane approach from the caudal edge of the probe and advanced until foramen is reached. After aspiration and confirmation of no intravascular injection, 1 ml of local anesthetics is administered^21,22^.

A previous case report has demonstrated the successful use of bilateral infraorbital nerve blocks combined with general anesthesia for better perioperative analgesia in a paediatric patient undergoing a transsphenoidal resection of a suprasellar tumour^23^.In one prospective randomized study, application of bilateral infraorbital block with 0.5% bupivacaine for transsphenoidal pituitary surgery has resulted in a significant increase for first demand to analgesia and significant decrease of analgesic consumption, whereas patient satisfaction for postoperative analgesia was found to be good^18^. Other regional techniques have also been attempted, which include bilateral sphenopalatine ganglion block^24^ and bilateral maxillary nerve blocks^25^. These have been shown to suppress the intraoperative sympathetic stimulation and hemodynamic responses during transsphenoidal surgeries.

#### 2.1.3 Trigeminal Nerve Block

Patients who are unresponsive to medical management of trigeminal neuralgia require injection of local anaesthetics^26^. Blockade of the branches of the trigeminal nerve (V2 and V3) in the pterygopalatine or infratemporal fossa have been traditionally performed using the paresthesia technique, by positioning the needle under fluoroscopy or computed tomography. The classical approach to access the Gasserian ganglion is via the foramen ovale. Usually, X-ray guided techniques, that rely on bony anatomical landmarks such as the maxilla, lateral pterygoid plate, and foramen ovale, are utilized. They can be difficult and often a challenge to interpret. Ultrasound guided needle placement allows real-time visualization of soft tissue and surrounding vasculature in addition to the appearance of bony structures. The image guidance permits delicate adjustment of the needle tip and direct observation of the injectate and, in doing so, confirm the local anesthetic spread at the intended region. The lateral pterygoid plate, the maxillary artery, and the pterygopalatine fossa can be easily ultrasonographically identified. The placement of the injectate anterior to the lateral pterygoid plate, below the lateral pterygoid muscle, can be visualized in real time. This approach allows access to the pterygopalatine fossa and its contents, including the sphenopalatine ganglion and the superficial and deep petrosal nerves^27^. In addition, as previously demonstrated using fluoroscopy, because the volume of the pterygopalatine fossa is small, placing 2 ml of contrast in this space produces a retrograde passage to reach the middle cranial fossa and allows visualization of the trigeminal ganglion^28^. Thus, ultrasound guidance makes it a safe and radiation free percutaneous procedure to provide sustained pain relief in patients unable to get relief by conservative measures from trigeminal neuralgia.

#### 2.1.4 Cervical plexus block (CPB)

Most common indication of deep and superficial cervical plexus block (CPB) in neurosurgery is carotid endarterectomy (CEA), in which an awake patient can self-monitor the sufficient cerebral blood flow throughout cross-clamping of the carotid artery. In recent years other indications of cervical plexus block is cervical spine surgery. For the superficial cervical plexus block, local anesthetic is superficially injected to the deep cervical fascia. In intermediate cervical plexus block, the injection is made between the investing layer of the deep cervical fascia and the prevertebral fascia, whereas for the deep cervical plexus block, local anesthetic is deposited deep to the prevertebral fascia.

The efficacy of superficial and deep cervical plexus block for CEA is the same. However, superficial CPB involves less complications. In addition, due to the possibility of phrenic nerve paresis, the deep cervical plexus block is relatively contraindicated in patients with contralateral phrenic nerve palsy and major pulmonary compromise^29,30^. Regardless of the mode of CPB used for CEA, there is a need of supplementation of local anesthetics to subcutaneous or deep tissues during surgery, because within the neck, there are few areas innervated by cranial nerves where even deep CPBs cannot reach^31-33^.

For performance of deep cervical block, the patient lies supine with head turned to opposite side. A line is drawn joining the mastoid process (MP) to the Chassagne tubercle (transverse process of the sixth cervical vertebra [C6]). When this line is sketched, the injection sites are marked over the C2, C3 and C4 which are situated on the MP–C6 line 2 cm, 4 cm, and 6 cm below the mastoid process respectively.

Palpating finger is placed just behind the posterior border of the sternocleidomastoid muscle. The needle is inserted in skin perpendicularly between the palpating fingers, and advanced until the transverse process is contacted. Now the needle is withdrawn 1–2 mm and 3-4 ml of local anesthetics is administered at each level once negative aspiration for blood is confirmed. In addition to CPB, CEA also needs blocking of the branches of glossopharyngeal nerve, which is performed by administrating the local anesthetic in the carotid sheath.

Superficial cervical plexus blockade performance requires the patient to be supine and his/her head turned to the opposite side. A line adjoining the mastoid process to C6 is drawn. The needle insertion is marked at the midpoint of this line, where the branches of the superficial cervical plexus arises from behind the posterior border of the sternocleidomastoid muscle. A total 10-15 ml of local anaesthetics is injected using a fan technique in 2-3 cm superior and 2-3 cm inferior direction of the needle (3–5 ml per each redirection/injection). The objective is to attain blockade of all four major branches of the superficial cervical plexus^34^.

CPB (superficial) can also be attempted under ultrasound guidance too. An ultrasound linear transducer (8–18 MHz) is placed on the lateral neck, overlying the sternocleidomastoid muscle at the level of the cricoid cartilage and then scanning is posteriorly done. The cervical plexus is appreciated as a small assembly of hypoechoic nodules (honeycomb appearance) just superficial to the prevertebral fascia that covers the interscalene groove. Now using either an in-plane or out-of-plane approach, block needle is inserted through the skin, platysma, and investing layer of the deep cervical fascia, and the needle tip is placed in proximity to the plexus. After negative aspiration, approximately 5–15 ml of local anesthetics is injected to envelop the plexus. In the case the plexus is not visualized, drug deposition deep to the sternocleidomastoid is usually sufficient to block plexus.

The biggest randomized trial until date on this topic is GALA trial^35^, which compared CEA under general anesthesia with that under local anesthesia. This trial revealed no difference in 30-day stroke or mortality rates. However, for patients with a contralateral carotid occlusion, local anesthetic techniques might offer some benefit, presumably related to its effect on preserving autoregulation and, therefore, blood flow to the contralateral hemisphere. However, the authors suggested that the trends revealing fewer peri-operative deaths and improved one-year survival following LA surgery requires further analysis.

In recent years other indications of cervical plexus block was suggested, namely cervical spine surgery. One study showed that preoperative superficial CPB is an effective approach for improving the initial quality of recovery in patients undergoing single- or two-level anterior cervical discectomy and fusion (ACDF). However, there was no influence on opioid consumption or discharge times^34^. In a different study it was determined that general anesthesia was superior to CPB for better intraoperative hemodynamic stability, providing high patient satisfaction with no intraoperative pain for patients undergoing ACDF. However, it results in longer surgery and anesthesia time, and it needs more postoperative analgesic and anesthesia cost^36^.

### 2.2. Blocks used for spinal surgeries

#### Erector Spinae Block (ESP)

Spinal surgery is common, and it varies from minimally invasive, single-level decompression to very complex, multi-stage extensive reconstruction^37^. In major spine surgery, patients experience intense and severe postoperative pain within the first 4 hours, which progressively decreases by the third day^38^. Pain following spinal surgery can originate from vertebrae, disks, ligaments, dura, facet joint, muscle, fascia, subcutaneous and cutaneous tissues^3^. Although, the majority of the spinal procedures are commonly performed under general anesthesia, sole regional anesthesia may be an option for one- or two-level lumbar laminectomy or disc surgery^39^.

The major advantages of regional anesthesia (spinal and epidural anesthesia, intrathecal injection of opioids and cervical plexus block) for spine surgery are hemodynamic stability, decreased postoperative pain, nausea vomiting and analgesic requirement in postoperative period^40-42^.

Epidural analgesia is considered as the gold standard for postoperative analgesia in lumbar spine surgeries, but the catheters may interfere with surgery. Also, there is chance of intrathecal penetration of local anesthetic if dura mater damage occurs during surgical procedure^43,44^.

In addition to the cervical CPB previously described for cervical spine surgeries, bilateral ultrasound guided erector spinae plane block, a relatively newer plane block, was first defined by Forero et al. in 2016. This block has been shown to have comparable analgesic properties to epidural block^45^. The site of ESPB administration is deep into the erector spinae muscle and superficial to the tips of the thoracic transverse processes, distant from the pleura and major blood vessels. Pneumothorax has been stated as one of the major complications of this procedure. ESP block has been used as successful postoperative analgesic treatment method in abdominal, thoracic, and breast surgeries^46-49^. Additionally, ESP blocks have also been used for postoperative analgesia in spinal surgery and published as case reports^41-43^.

The erector spinae include several muscles, such as the iliocostalis, longissimus, and spinalis muscles. They extend bilaterally from skull to sacral region longitudinally and from spinous to transverse process up to ribs horizontally. This block is performed post induction, after making patient prone, although it can also be performed in a sitting or lateral decubitus positions. As with the majority of the other plane blocks, this block is also performed under ultrasound guidance. After strict aseptic precaution, a high-frequency (10–15 MHz) linear-array ultrasound transducer (offers a higher-resolution image) is needed, although a low-frequency curvilinear probe is beneficial in obese patients where the transverse processes is situated at a depth more than 4 cm. To visualize the transverse process, the transducer is kept about 3 cm lateral to the spinous processes in a longitudinal parasagittal alignment. Transverse process is identified as flat, squared-off acoustic shadows, whereas ribs are visualized as rounded acoustic shadows (if the transducer is placed too laterally) and thoracic laminae are identified as flat hyperechoic lines (if the transducer is placed too medial). After correct identification of the transverse process, an 18-gauge echogenic needle is inserted with an in-plane, cranial-to-caudad direction to touch the bony shadow of the transverse process by the tip deep to the fascial plane of the erector spinae muscle. The accurate location of the needle tip is established by administrating 1-2 ml of normal saline and noticing spread of fluid lifting the erector spinae muscle above transverse process. Now, either longer acting local anesthetics can be administered in bolus dose or a catheter can be placed through the needle for continuous drug infusion, for longer duration. The simple aspects in the acceptance of this block are easy sonographic identification of landmarks and a minor complication ratio compared to the paravertebral block and its alternatives^50^. The exact mechanism of action is not fully known. An interfascial spread toward the dorsal rami of spinal nerves is possibly the key mechanisms of action. These rami carry visceral motor, somatic motor, and sensory information to and from the skin and deep muscles of the back. The extent of the block and its analgesic effect is variable due to the variability in the craniocaudal spread of the drug^50^. Different studies compared ESP block group with control group for post-operative analgesia in patients undergoing elective lumber surgery and concluded that there was decreased pain score at different time points after surgery, decreased 24-hour postoperative cumulative opioid requirements, time to first analgesic requirement was significantly longer, as well as more favourable patient satisfaction scores in ESP group of patients^51,52^. Another retrospective study concluded that the ESP block for patients undergoing lumbar laminoplasty provides more effective analgesia, which persists until the morning of the second postoperative day^53^.

**Table 1:** Table 1. Summary of studies related to regional nerve blocks used in neurosurgery RCT – randomised control study

Study	Type of study	Type of surgery	Type of regional nerve block	Intervention	Findings
Gazoni FM et al.^5^ 2008	RCT	Supratentorial craniotomy	Scalp block	14 patients (received a skull block with 0.5% ropivacaine at least 15 minutes prior to pinning) vs. 16 patients (no skull block).	Significant hemodynamic (heart rate) stability at the time of head pins in the skull block group.
Pinosky ML et al.^6^ 1996	RCT	Supratentorial craniotomy	Scalp block	21 patients were allocated in study group (skull block with 0.5% bupivacaine) vs. control group (skull block with normal saline) after a standardized induction and 5 min prior to head pinning.	Significant hemodynamic (blood pressure, heart rate) stability at the time of head pins in the skull block group.
Geze S et al.^7^ 2009	RCT	Supratentorial craniotomy	Scalp block	45 patients were allocated in group L (0.5% bupivacaine was infiltrated at each pin-insertion site) vs. group S (skull block with 0.5% bupivacaine), 5 minutes before head pinning vs. control group (opioids were used to control hemodynamic responses).	Significant hemodynamic (blood pressure, heart rate) stability at the time of head pins in the skull block group during head pinning and also at the 1st, 2nd and 3rd minutes after pinning. Plasma cortisol and adrenocorticotropic hormone levels measured at the 5th and 60th minutes after pinning were significantly lower in group S than those in groups L and C.
Lee EJ et al.^8^ 2006	RCT	Supratentorial craniotomy	Scalp block	16 patients were randomized in study group (skull block with 0.25% bupivacaine) vs. control group (skull block with normal saline).	Significant hemodynamic (blood pressure, heart rate) stability during the surgical period between incision and dural opening in study group. However, plasma catecholamine metabolites were comparable in both groups.
Bala et al.^9^ 2006	RCT	Supratentorial craniotomy	Scalp block	40 patients were randomized in study group (skull block with 0.5% bupivacaine with 1:400,000 adrenaline) vs. control group (skull block with normal saline with 1:400,000 adrenaline), both after skin closure.	Significant decrease in incidence and severity of postoperative pain, as well as a decrease in analgesic requirement in scalp nerve block group of patients.
Nguyen A et al.^10^ 2001	RCT	Supratentorial craniotomy	Scalp block	30 patients were randomly divided into two groups: ropivacaine (scalp block with ropivacaine 0.75%) and saline (scalp block with 0.9% saline).	Postoperative scalp block decreases the severity of pain after craniotomy 4, 8, 12, 16, 20, 24, and 48 h.
Jose R et al.^12^ 2017	RCT	Supratentorial craniotomy	Scalp block	90 patients were randomized in study group (skull block with local anesthetics and 8 mg of dexamethasone) vs. control group (skull block with local anesthetics and 2 ml of normal saline) soon after induction of general anesthesia.	Addition of dexamethasone as an adjuvant to local anesthetics in scalp nerve blocks in the setting of perioperative steroid therapy does not appear to provide any additional benefit with respect to prolongation of the duration of the block
Yasser M. et al.^13^ 2020	RCT	Awake craniotomy	Scalp block	40 patients were randomly allocated into 4 equal scalp block groups. Group I (bupivacaine 0.25%+lidocaine 1% with 1:200,000 epinephrine) Group II (same as Group I + 8 mg dexamethasone) Group III (same as Group I + 500 mgMgSO4) Group IV (same as Group I + 8 mg dexamethasone + 500 mgMgSO4).	Use of either 8 mg dexamethasone, or 500 mg MgSO4, or both, as adjuvant to bupivacaine-lidocaine for scalp block improves efficacy of the block.
Guilfoyle MR et al.^14^ 2013	Meta-analysis	Supratentorial craniotomy	Scalp block	7 RCTs with a total recruitment of 320 patients evaluating the effect of regional scalp block on postoperative pain after craniotomy.	Meta-analysis shows a consistent finding of reduced postoperative pain.
Wardhana A et al.^15^ 2019	Meta-analysis	Supratentorial craniotomy	Scalp block	A total of 10 RCTs (551 patients) evaluating the effect of scalp block on post craniotomy pain compared to no-scalp block.	Scalp block might be effective at <6 h post craniotomy with very-low quality evidence. It also shows moderate effect on reducing total 24 h opioid consumption.
Ayoub C et al.^16^ 2006	RCT	Supratentorial craniotomy	Scalp block	Fifty craniotomy patients were randomized into two groups: morphine (morphine 0.1 mg/kg IV after dural closure and scalp block with 0.9% saline at the end of surgery) and block (0.9% saline after dural closure and scalp block with a 1:1 mixture of bupivacaine 0.5% and lidocaine 2% at the end of surgery).	Quality of analgesia and postoperative hemodynamic profile was similar in both groups.
Jonnavithula N et al.^18^ 2011	RCT	Transsphenoidal hypophysectomy	Infraorbital block	20 patients were randomized into two groups: group I (bilateral infraorbital block with 0.5% bupivacaine) vs group II (no block).	In addition to postoperative analgesia infraorbital block useful in providing rapid, smooth, pain free emergence from anesthesia, facilitating prompt neurological assessment; provides effective postoperative analgesia and relieves discomfort of nasal packing.
Mariano ER et al.^19^ 2009	RCT	Nasal surgery	Infraorbital block	40 patients were randomly assigned to receive bilateral infraorbital injections with either 0.5% bupivacaine (group IOB) or normal saline (group NS) using an intraoral technique.	Compared to group NS, subjects in group IOB did experience a reduction in postoperative pain.
McAdam D et al.^23^ 2005	Case Report	Transsphenoidal hypophysectomy	Infraorbital block	Bilateral infraorbital nerve blocks were performed using an intraoral approach in an 11-year old after induction and repeated at the conclusion of surgery.	Patient didn’t need additional analgesia in postoperative period.
Wang H et al. ^34^ 2017	RCT	Anterior cervical discectomy and fusion (ACDF)	Cervical plexus block	356 patients who underwent 1-level ACDF for cervical spinal myelopathy were assigned to receive general anesthesia (group A) and cervical plexus block (group B).	Group A patients had better intraoperative hemodynamic stability with no intraoperative pain. However, it requires longer surgery and anesthesia time, and need more postoperative analgesic & anesthesia cost.
Lewis SC et al.^35^ (GALA TRIAL) 2008	Multicenter RCT	Carotid endarterectomy	Cervical plexus block	Multicenter RCT of 3526 patients with symptomatic or asymptomatic carotid stenosis from 95 centers in 24 countries. Participants were randomly assigned in equal number to carotid endarterectomy under general or local anesthesia.	The two groups did not significantly differ for quality of life, length of hospital stay in the prespecified subgroups of age, contralateral carotid occlusion, and baseline surgical risk.
Mariappan et al ^36^ 2015	RCT	Anterior cervical discectomy and fusion (ACDF)	Cervical plexus block	46 patients were randomized to receive either a superficial cervical plexus block (SCPB) (0.25% bupivacaine, 10 mL) or no block for elective single- or two-level ACDF.	Preoperative SCPB is an effective strategy for improving the early quality of recovery in patients undergoing single- or two-level ACDF.
Melvin JP et al.^49^ 2018	Case series	Lumbosacral spine surgery, lumbar decompressions, sacral laminoplasties & coccygectomy	Erector spinae block (ESP)	Bilateral erector spinae block blocks at the T10 or T12 level in six patients.	All patients had minimal postoperative pain and very low postoperative opioid requirements.
Yayik AM et al.^52^ 2019	RCT	Lumbar decompression surgery	Erector spinae block	60 patients undergoing open lumbar decompression surgery were randomly assigned to 2 groups. The ESP group received ultrasound guided bilateral erector spinae block with 0.25% bupivacaine. In the control group, no intervention was performed.	Significant postoperative pain control, decreased analgesic requirement and increased time to first analgesic requirement in ESP group.
Ueshima H et al.^53^ 2019	Retrospective study	Lumbar spinal surgery	Erector spinae block	41 patients undergoing lumbar spinal surgery were retrospectively analyzed. Of these, 23 received only general anesthesia (G group), whereas the other 18 patients received the ESP block in addition to general anesthesia (E group).	The ESP block provides effective postoperative analgesic effect for 24 hours in patients undergoing lumbar spinal surgery.
Singh et al.^54^ 2019	RCT	Lumbar spine surgery	Erector spinae block	40 adults were randomly assigned to the control group-no preoperative ESP block, or ESP block group-preoperative bilateral ultrasound-guided ESP block.	Significant decrease in postoperative pain control and analgesic requirement in ESP block group. Patient satisfaction scores were more favourable within the block group.

## 3. Conclusion

Application of regional anesthetic techniques in the domain of neurosurgery had conventionally been an overlooked clinical area. Perioperative pain management in neurosurgical patients has been traditionally reliant on intravenous and oral analgesics. Apart from the reasons previously mentioned, regional techniques had concerns of motor blockade, failure and infection, which restricted their widespread usage. However, better visualization with ultrasound, refinements in techniques and development of LA with lower propensity of motor blockade has revitalized their role in intraoperative management neurosurgical procedures and the postoperative pain, which has been usually inconsistently recognized and inadequately treated. Simultaneously, an increased awareness of pain management in general, along with advances in understanding of pain modulation and pathophysiology, has led to improved practice and perioperative care of patients, leading to resurgence in regional techniques. This has resulted in better operative conditions and parameters, along with an increased patient satisfaction, in terms of analgesia, mobility and early recovery. Since the area is still relatively unexplored, with a vast potential for improvements in terms of new strategies and techniques, it is worthwhile to undertake further studies and investigations in this regard.

## KEY POINTS

**◊** **Regional techniques in neuroanesthetic practice have not been widely explored**

**◊** **Refinements in terms of techniques and drugs have increased their utilization in neurosurgical practices, for smoother intraoperative course and improved postoperative patient comfort**
